# Evaluation of Hand Hygiene Technique in Uzbekistan: First Experience from Semmelweis Scanner-Based Digital Assessment in Educational Institutions

**DOI:** 10.3390/healthcare14111474

**Published:** 2026-05-26

**Authors:** Shavkat Azimov, Peter Szeremy, Sherzod Nematov, Jamoliddin Razzokov, Temurbek Daminov, Durdona Rasulova, Tamás Haidegger

**Affiliations:** 1 Department of Biotechnology, Tashkent State Technical University, Universitet 2, Tashkent 100095, Uzbekistan; 2 University Research and Innovation Centre (EKIK), Obuda University, H-1034 Budapest, Hungary; haidegger@irob.uni-obuda.hu; 3 Department of Natural Sciences, Karshi State Technical University, Mustaqillik Avenue Street 225, Karshi 180100, Uzbekistan; 4 Institute of Fundamental and Applied Research, National Research University TIIAME, Kori Niyoziy 39, Tashkent 100000, Uzbekistan

**Keywords:** hand hygiene technique, Semmelweis system, digital monitoring, infection prevention

## Abstract

**Highlights:**

**What are the main findings?**

- A total of 4191 hand hygiene scans were analyzed using the Semmelweis digital assessment system.
- Only 43.4% of measurements achieved the ≥95% hand coverage threshold, with thumb bases and fingertips being the most frequently missed regions.

**What are the implications of the main findings?**

- The high rate of missed areas highlights a critical gap in standard hand hygiene compliance.
- Scanner-based visual feedback can be implemented to support objective, technique-focused hand hygiene education.

**Abstract:**

**Background**: Hand Hygiene (HH) plays a crucial role in preventing Hospital-Acquired Infections (HAIs), yet compliance and technique often remain inadequate. To our knowledge, this study is among the first large-scale Semmelweis Scanner-based evaluations of hand hygiene technique among both medical and non-medical students in Uzbekistan and the wider Central Asian region. **Methods**: A cross-sectional study was conducted between March 2024 and July 2025 at the Tashkent Medical Academy and the Tashkent State Technical University, resulting in 4191 data scans and 16,764 pictures analyzed. Hand surface coverage was evaluated using the Semmelweis digital monitoring system, which provides image-based feedback on insufficiently covered areas. Adequate performance was defined as achieving at least 95% hand surface coverage. **Results**: The findings showed that only 43.4% of hand hygiene measurements achieved the ≥95% hand coverage threshold, while 56.6% showed incomplete coverage. The sixth WHO-recommended step, i.e., rotational rubbing of the thumb and fingertips was identified as the most frequently missed moment. Significant variation was observed across faculties and departments, with bachelors achieving the highest success (*n* = 1012, 51.8%) and Ph.D. students reaching the lowest (18.4%). **Conclusions**: Hand hygiene technique among students in Uzbekistan is highly variable and frequently inadequate when evaluated using scanner-based digital assessment. The findings suggest that incomplete hand surface coverage, rather than the absence of sanitization attempts, is the principal challenge. Digital hand hygiene monitoring systems can provide an effective complementary tool for technique-focused education and can support infection prevention practices in both medical and non-medical training environments.

## 1. Introduction

Infectious diseases remain one of the leading causes of morbidity and mortality worldwide, posing an ongoing challenge to healthcare systems and societies alike [1,2]. Despite remarkable advancements in modern medicine, including the development of vaccines, antibiotics, and improved surgical techniques, the human race continues to face the persistent and evolving threat of pathogenic microorganisms [3,4,5]. Among the strategies employed to combat infections, antimicrobial agents, particularly antibiotics, have historically played a central role in reducing infection-related mortality. However, the escalating global crisis of antimicrobial resistance (AMR) has severely undermined the effectiveness of conventional therapies [6,7,8]. According to the World Health Organization (WHO), if urgent action is not taken, AMR could lead to 10 million deaths annually by 2050 and an estimated economic loss of 100 trillion USD globally [9]. As a result, there has been a paradigm shift toward prevention-based strategies, where improving hygiene practices plays a pivotal role in reducing infection transmission in both community and healthcare settings [10,11].

Among various preventive measures, hand hygiene is widely recognized as the first line of defense against the spread of infections [12]. The transmission of pathogens—including multidrug-resistant organisms—commonly occurs via contaminated hands of healthcare workers, medical students, and patients. Studies have consistently shown that up to 80% of Hospital-Acquired Infections (HAIs) can be traced back to poor compliance with hand hygiene guidelines [13,14]. HAIs affect hundreds of millions of patients globally each year, resulting in significant clinical and economic burdens. According to WHO estimates, approximately 7 million HAIs occur annually in high-income countries, while the burden is significantly higher in low- and middle-income states, affecting up to 15% of hospitalized patients [15]. Inadequate hand hygiene practices are responsible for a substantial proportion of postoperative wound infections, bloodstream infections, ventilator-associated pneumonias, and urinary tract infections [16].

The consequences of HAIs are profound, leading not only to increased morbidity and mortality but also to prolonged hospital stays, greater antibiotic usage, and increased healthcare costs. Postoperative infections, in particular, account for a significant proportion of preventable deaths and impose an enormous financial burden on healthcare systems [17]. In resource-limited settings, where access to advanced sterilization techniques and broad-spectrum antimicrobials is restricted, the importance of effective hand hygiene is even more pronounced [18]. As new resistant pathogens continue to emerge, hospitals face growing pressure to adopt innovative technological solutions to ensure compliance with infection control protocols [19].

Traditional strategies to improve hand hygiene compliance—such as awareness campaigns, posters, and manual audits—have proven insufficient in maintaining long-term behavioral change among healthcare workers [20]. Consequently, the integration of digital technologies has emerged as a promising approach to address these limitations. Among these technologies, hand hygiene scanners represent a particularly innovative solution, providing real-time feedback on the quality and completeness of hand sanitization technique [21]. These devices utilize advanced optical sensors, UV fluorescence, or multispectral imaging techniques to detect residual contaminants and highlight areas commonly missed during washing, namely thumbs, fingertips, interdigital spaces, and wrists [22,23,24]. By enabling objective assessment and immediate corrective action, hand scanners enhance self-awareness and educational effectiveness of both medical staff and students.

In this study, we employed the Semmelweis System, an advanced hand hygiene monitoring and education device. The scanner provides an objective, quantitative evaluation of hand rubbing and hand washing technique by using fluorescently labeled training material and Artificial Intelligence (AI)-based image analysis to visualize areas missed during hand hygiene procedures. While the application of AI has several limitations, the current practice has already proved itself through hundreds of thousands of measurements [25,26]. Our study was conducted among students from two institutions—Tashkent Medical Academy and Tashkent State Technical University—to evaluate their hand hygiene practices, a first of such a large-scale assessment study in the region, to the knowledge of the authors. The selection of participants from both medical and non-medical faculties allowed us to compare hygiene knowledge and behavioral patterns through different educational backgrounds.

## 2. Methods

*Study Design and Participants*: This cross-sectional study was carried out between 19 March 2024 and 23 July 2025, utilizing data obtained from the Semmelweis Hand Hygiene System (HandInScan Zrt., Debrecen, Hungary) at two locations: Tashkent Medical Academy and Tashkent State Technical University, Uzbekistan. The study population consisted of students from both institutions, inclusion was on a voluntary basis, independently from any clinical work. Users could perform multiple measurements during the test period. The system stored anonymized educational scan records, and repeated use of the scanner by the same individual was possible during the study period. Because persistent participant identifiers were not retained in the exported research dataset, the exact number of unique participants could not be reconstructed retrospectively. Accordingly, the present analysis is reported at the scan level rather than the participant level, and inferential findings should be interpreted with appropriate caution.

*Hand Scanner Technology*: The study utilized the Semmelweis System, an advanced digital imaging device designed to assess the effectiveness of hand hygiene procedure as measured by skin surface coverage. The scanner operates by combining fluorescent markers with high-resolution digital imaging and AI-based evaluation to visualize areas where sanitizer application is insufficient. Participants were instructed to apply 3 mL of Semmelweis Training Rub, a UV-fluorescent training formulation supplied with the hand hygiene assessment system, and to perform hand rubbing according to their current knowledge of the standard hospital-recommended technique. Before scanning, participants received brief verbal instructions on the procedure and were informed that the scanner would assess the completeness of hand surface coverage under UV-A illumination. Digital images of the palmar and dorsal hand surfaces were then captured, and a color-coded heatmap was automatically generated to identify insufficiently covered areas. A color-coded heatmap was automatically generated, highlighting the missed or insufficiently covered areas such as the thumbs, fingertips, interdigital spaces and wrists. All images and quantitative data were securely stored in the Semmelweis System and used exclusively for educational and research purposes.

*Data Collection and Analysis*: Scanner-generated logs collected throughout the study period, which spanned over one year, were systematically retrieved and analyzed. For each participant, hand coverage was quantified based on the percentage of hand surface effectively covered with the training product. The scanner automatically identified and recorded commonly the missed regions. For statistical evaluation, data were categorized based on institution, faculty, age group, and gender. Descriptive statistics were calculated to summarize coverage results, while independent *t*-tests were used to compare mean coverage between groups. Additionally, chi-square tests were performed to evaluate differences in categorical variables, such as the frequency of missed areas. A *p*-value < 0.05 was considered statistically significant. The ≥95% hand coverage threshold was used as the primary criterion for adequate performance because this cut-off has been widely used in Semmelweis scanner-based educational practice and provides a stringent benchmark for near-complete coverage. To test the robustness of the findings, we also performed a sensitivity analysis using alternative thresholds of 90% and 98%.

*Ethics and data governance*: The study was conducted within the framework of the project “Evidence-Based Digital Healthcare Solutions Supporting Local Patient Safety Procedures,” implemented at Tashkent State Technical University in collaboration with Tashkent Medical Academy. The hand hygiene experiments at Tashkent Medical Academy were performed under official institutional cooperation according to the directive of Tashkent State Technical University dated 27 March 2024 (Ref. No. 01/9-14-770), and the protocol received a positive conclusion from the Scientific and Technical Council of the Academy.

The study involved non-invasive educational assessment of hand hygiene technique. Participants were informed through official university communication prior to participation, and participation was voluntary. No facial images, biometric identifiers, or clinical data were collected. Scan data were used exclusively for educational and research purposes. Depending on the export setting, records were either pseudonymized for repeated educational tracking or anonymized for final analysis.

## 3. Results and Discussion

A total of 4191 scans were taken and 16,764 pictures were analyzed from the Tashkent Medical Academy (Tashkent, Uzbekistan) and the Tashkent State Technical University (Tashkent, Uzbekistan) during the study. Rings were detected in 2280 of 4191 scans (54.4%), indicating that jewelry use during hand hygiene assessment was common in the study population. Because rings may interfere with complete hand coverage, this observation highlights a potentially important practical barrier that warrants focused investigation in future studies. The presence of rings is a critical factor, as numerous studies have demonstrated that rings, bracelets, and artificial nails can hinder effective sanitizer coverage and increase bacterial colonization [27,28,29].

**Figure 1:**
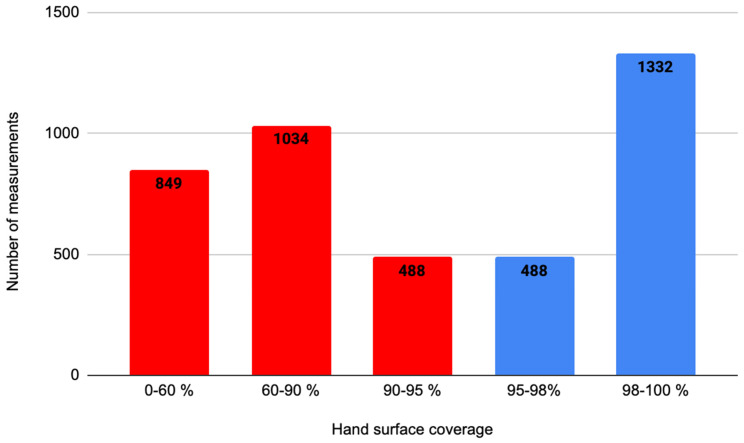
Distribution of hand hygiene scan results among all participants. The success rate is represented in blue and indicates sufficient sanitizer coverage. Failure rates (in red) corresponded to inadequate coverage levels.

The analysis of 4191 total scans revealed substantial variation in hand sanitizer coverage among participants (Figure 1). A significant proportion of scans (~56.6%) resulted in insufficient sanitizer coverage, falling beneath the 95% (clinically acceptable) threshold. Only ~43.4% of scans achieved satisfactory results. Low-coverage measurements (0–60%) accounted for 20.3%, while 60–90% contributed the remaining quarter (24.7%). The 90–95% range took another 11.6%. Measurements at or above 95% coverage represented 43.4% of the dataset. Within this high-performance group, 95–98% accounted for 11.6%, while 98–100% alone constituted 31.8% (Table 1). To assess whether the main findings depended on the selected ≥95% coverage threshold, an additional sensitivity analysis was performed using alternative cutoffs of 90% and 98%. Although absolute pass rates changed, the overall ranking pattern across student groups remained similar, indicating that the principal findings were robust to threshold selection.

In Table 1, the rows labeled “<95% total” and “≥95% total” represent cumulative summary categories based on the predefined threshold and are presented in addition to the detailed coverage intervals. These findings indicate that despite repeated a priori educational events, a considerable number of participants failed to achieve optimal hand coverage and sanitization on the first try. This highlights a knowledge-to-practice gap in proper hand hygiene techniques, among students, and emphasizes the need for continuous training and feedback mechanisms. An important limitation of this study is that the exported research dataset was available at the scan level, whereas repeated scans by the same participant were possible during the study period. Because persistent participant identifiers were not retained in the final anonymized export, the exact number of unique participants could not be reconstructed retrospectively. This limits participant-level inference and may have influenced the assumptions of independence underlying some statistical tests.

**Figure 2:**
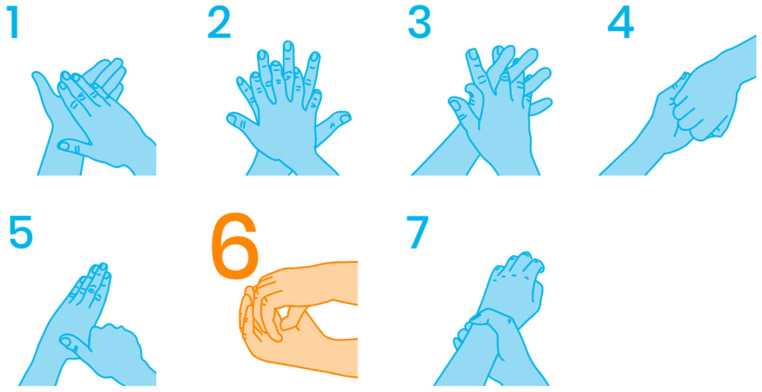
Analysis of missed steps and areas during hand hygiene procedures. The sixth step of the WHO-recommended hand hygiene protocol—rotational rubbing of the thumb and fingertips—was identified as the most frequently missed step among participants.

Analysis of scanner-generated *most frequently missed area* heatmaps revealed that the sixth step of the WHO’s 6-step hand hygiene protocol [30], i.e., rotational rubbing of the thumb and fingertips was the most frequently omitted step among participants (Figure 2). This omission significantly impacted overall skin coverage, particularly around the thumb bases and fingertip regions, which are known to harbor high microbial loads if not adequately cleaned [31].

**Figure 3:**
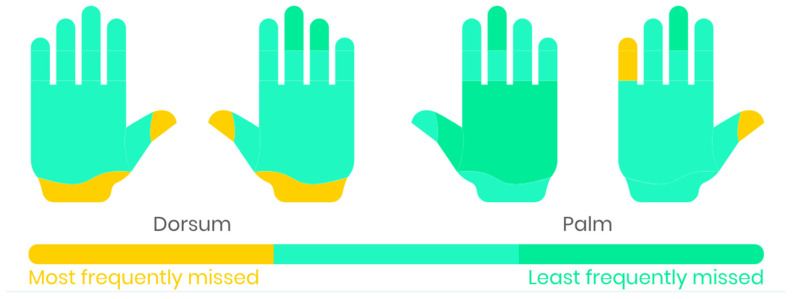
Heatmap representation of the most commonly missed regions on both the dorsum and palm surfaces of the hands. Critical zones, including thumb bases, fingertips, and interdigital spaces, were most frequently neglected, while central palm areas showed higher sanitizer coverage.

Figure 3 illustrates the distribution of missed areas across both the dorsal and palmar hand surfaces. The most neglected regions included: thumb bases (dorsal and palmar sides), fingertips, interdigital spaces, lateral edges of the hands, which is in line with other international studies’ findings [25]. In contrast, the central palm regions demonstrated higher sanitizer coverage, suggesting that participants focused primarily on easily accessible areas while overlooking anatomically challenging zones.

Our findings highlight significant technique-related gaps in hand hygiene practices, even among highly educated participants, including medical students. Although sanitizer was applied at adequate quantities [10], critical infection-prone regions—notably the thumb bases and fingertips—were frequently missed. These areas are recognized as microbial hotspots due to direct patient contact, making their omission particularly concerning in clinical settings. The omission of the sixth WHO-recommended step reflects inadequate integration of proper technique into routine hygiene behavior. Similar results have been reported in earlier studies, which found that rotational thumb rubbing and fingertip cleaning are among the least-followed hand hygiene steps globally. This suggests a behavioral gap rather than a lack of awareness, emphasizing the need for practical, visual, and feedback-based training.

The results demonstrate pronounced differences in hand hygiene performance across student groups. Across all groups, 4191 measurements were evaluated, including 1819 passes (43.4%) and 2372 failures (56.6%). Bachelor students contributed the largest number of successful scans (*n* = 1011), corresponding to 55.6% of all successful measurements. Importantly, Bachelor students showed the highest mean hand coverage (84.0%), whereas their pass rate at the ≥95% threshold was 51.8%. This distinction between threshold-based pass rate and mean hand coverage has now been clarified throughout the text and tables. Master students represented the second largest contribution (17.8% of passes), followed by surgery students (7.9%), while all other groups individually contributed less than 6% to total successes. Despite smaller absolute numbers, nursing and surgeon students showed relatively high success rates (81.5% and 78.6%, respectively), whereas dentistry, PhD and surgery students exhibited notably lower performance, with surgery students showing the lowest success rate (57.8%) and the highest failure count (435 cases). A chi-square test of independence demonstrated a statistically significant association between student group and hand coverage outcome (χ^2^ = 175.6, *p* < 0.001), indicating that success is not evenly distributed across groups.

All groups show lower odds of achieving adequate hand coverage compared to Bachelor students, with the strongest reduction observed in surgery (OR ≈ 0.31) and PhD students (OR ≈ 0.21). With the χ^2^ test (*p* < 0.001), this supports that training level and educational focus are strongly associated with hand coverage performance, even within an exclusively student population (Table 2).

The results demonstrate pronounced differences in hand hygiene performance across student groups. Bachelor students had the highest mean coverage (84.0%) and the highest pass rate at the ≥95% threshold (51.8%). Nursing and Master’s students also showed relatively high mean coverage values (81.5% and 80.8%, respectively), whereas surgery, dentistry, and PhD groups demonstrated lower performance overall.

In contrast, students enrolled in more specialized or clinically demanding fields exhibited reduced success rates. Surgeon and medical prevention students achieved moderate performance (78.6% and 74.7%, respectively), suggesting that while hygiene knowledge is present, competing task complexity and procedural focus may detract from optimal coverage. Therapy and dentistry students showed further declines (73.6% and 67.6%), a concerning finding given their regular patient contact, which points to a potential underemphasis of detailed hand coverage training within these curricula. The lowest performance was observed among surgery students (57.8%) and PhD students (69.6%), likely reflecting a stronger focus on technical procedures or research activities rather than routine clinical hygiene practices.

The present study provides one of the first large-scale, scanner-based evaluations of hand hygiene technique among student populations in Uzbekistan, revealing substantial gaps in effective hand surface coverage. Despite widespread awareness of hand hygiene importance, more than half of all scans failed to reach the ≥95% coverage threshold, underscoring a persistent discrepancy between theoretical knowledge and practical execution. The high proportion of low- and medium-coverage results suggests that inadequate technique, rather than lack of sanitizer use, is the primary limiting factor.

Marked differences were observed through student groups, indicating that educational focus and training context strongly influence hand hygiene performance. Bachelor and nursing students achieved the highest success rates, likely reflecting structured supervision and repeated reinforcement of basic hygiene protocols during early training stages. In contrast, students in more specialized or advanced programs, particularly surgery, dentistry, and PhD tracks, demonstrated significantly lower success rates and reduced odds of achieving adequate coverage.

The frequent omission of the sixth WHO-recommended step—rotational rubbing of the thumb and fingertips—is consistent with previous international findings and highlights a global behavioral pattern rather than a local anomaly. These anatomically complex regions are critical reservoirs for microbial transmission, making their neglect particularly concerning in clinical environments. The observed association between jewelry use and incomplete coverage further reinforces the need for targeted education addressing practical barriers to effective hand hygiene.

These findings are also consistent with previous studies using the same or closely related scanner-based assessment tools [32]. In an Italian neurological hospital, automated HandInScan^®^ monitoring demonstrated that baseline hand coverage among healthcare employees was suboptimal, with mean overall coverage of 74.5%, and that repeated assessments with feedback improved this value to 94.5%. Importantly, that study also showed that hand-back coverage remained lower than palm coverage throughout the observation period, confirming that anatomically more difficult regions tend to be insufficiently disinfected even in professional healthcare environments. Similarly, earlier Semmelweis Scanner–based studies and student-focused investigations identified the thumbs, fingertips, and dorsal regions as the most frequently missed areas during hand hygiene procedures. In this context, our results from Uzbekistan align well with the broader international literature, while extending the evidence to a wider educational setting that includes both medical and non-medical students in a Central Asian country.

Taken together, these similarities strengthen the interpretation of our findings and support the broader relevance of scanner-based educational feedback. Although the institutional settings differ between hospital employees and university students, the recurrent pattern of insufficient coverage in anatomically complex hand regions suggests that the challenge is fundamentally technique-related and not confined to a single professional or geographic context. This reinforces the potential value of digital hand hygiene monitoring systems as a practical educational tool in both developed and developing countries.

This study has several limitations. First, the dataset was collected in an educational setting and may not be fully generalizable to practicing healthcare workers or hospital workflows. Second, volunteer-based participation may have introduced selection bias. Third, depending on the export configuration, repeated scans by the same participant may have affected assumptions of statistical independence. Fourth, scanner-based hand coverage reflects technique quality rather than direct microbial reduction or infection outcomes. Finally, as with any AI-assisted imaging system, classification performance may depend on image quality, hand positioning, and the specific training characteristics of the device. These limitations should be considered when interpreting the results and planning future prospective or intervention-based studies.

Overall, the results demonstrated that traditional education alone is insufficient to ensure proper technique. Objective, visual feedback provided by digital hand hygiene monitoring systems appears essential for closing the knowledge-to-practice gap and fostering lasting behavioral change.

## 4. Conclusions

This study shows that scanner-based assessment can reveal substantial and systematic gaps in hand hygiene technique among university students in Uzbekistan. Although hand sanitization was routinely attempted, fewer than half of all scans achieved the ≥95% hand coverage threshold, indicating that incomplete surface coverage remains a common challenge. The most frequently missed regions were the thumb bases, fingertips, and interdigital areas, underscoring the need for technique-focused training.

Differences across student groups suggest that educational context, curricular emphasis, and repeated practical reinforcement may influence hand hygiene performance. Rather than replacing conventional teaching, digital hand hygiene monitoring systems may serve as a useful complementary tool within university curricula, simulation training, and clinical onboarding programs. Future studies should incorporate participant-level longitudinal designs and intervention-based evaluation to determine how scanner-based feedback can best support sustained improvement.

In conclusion, scanner-based assessment represents a powerful tool for strengthening hand hygiene education, promoting behavioral change, and supporting infection prevention strategies, particularly in resource-limited and educational settings.

## Figures and Tables

**Table 1:** Distribution of hand surface coverage categories across all measurements. Detailed coverage intervals are shown together with the cumulative summary categories below and above the predefined 95% threshold.

Hand Surface Coverage Category	Number of Measurements	Relative Frequency (%)	Cumulative Frequency (%)
0–60%	849	20.3	20.3
60–90%	1034	24.7	44.9
90–95%	488	11.6	56.6
<95% total	2371	56.6	56.6
95–98%	488	11.6	68.2
98–100%	1332	31.8	100.0
≥95% total	1820	43.4	100.0

**Table 2:** Group-wise hand hygiene outcomes based on scanner-assessed surface coverage. Pass rate at ≥95% (%) denotes the proportion of scans that met the predefined adequate hand coverage threshold. Mean hand coverage (%) denotes the average hand surface coverage across all scans within each student group. Odds ratios (ORs) are shown relative to Bachelor students.

Group	Pass Rate at ≥95% (%)	Mean Hand Coverage (%)	Passed	Failed	Total	Share of All Passes (%)	Odds Ratio (vs. Bachelor)
Bachelor	51.8	84	1011	941	1952	55.6	1.00 (ref.)
Masters	46.6	80.8	324	371	695	17.8	0.81
Nurses	41.7	81.5	40	56	96	2.2	0.67
Surgeons	40.3	78.6	50	74	124	2.7	0.63
Extra card	40.0	72.7	22	33	55	1.2	0.62
Medical prevention	35.5	74.7	22	40	62	1.2	0.51
Dentistry	34.6	67.6	101	191	292	5.6	0.49
Therapy	33.4	73.6	96	191	287	5.3	0.47
Surgery	24.9	57.8	144	435	579	7.9	0.31
PhD	18.4	69.6	9	40	49	0.5	0.21
Total	—	—	1819	2372	4191	100	—

## Data Availability

All data generated or analyzed during this study are included in this published article.
